# Disaster Caregiving: Caregivers Negotiating Storms and COVID-19 for Veterans and Non-Veterans on the Gulf Coast

**DOI:** 10.1093/geroni/igab046.1736

**Published:** 2021-12-17

**Authors:** Nathan Boucher, Kevin McKenna, Carrie Dombeck, Amy Clark, Ke Wang, Jennifer Olson, Megan Shepherd-Banigan

**Affiliations:** 1 Durham VA Health Care System, Durham, North Carolina, United States; 2 Duke University School of Medicine, Durham, North Carolina, United States; 3 Rosalynn Carter Institute for Caregivers, Americus, Georgia, United States; 4 Rosalynn Carter Institute for Caregiving, Americus, Georgia, United States

## Abstract

Natural disasters and COVID-19 likely add complexity to caregiving efforts, yet little is known about these effects. We will discuss our findings exploring additional needs and challenges experienced by caregivers during hurricanes, floods, and COVID focused on US Gulf Coast states. We interviewed caregivers of both Veterans (n=13) and non-Veterans (n=11). The presentation will include an overview of 1) types of resources needed or used related to storms and to COVID, including social support and access to information for both emergency planning and recovery; 2) caregiver experience before, during, and after the disaster including psychological effects on caregivers and addressing special health needs; 3) comparisons of challenges during storms versus COVID including emotional impact and access to health and specialty care; and 4) additional resources used by caregivers of Veterans. We will also address how these data are informing national caregiver support programs.

